# Miniaturization re-establishes symmetry in the wing folding patterns of featherwing beetles

**DOI:** 10.1038/s41598-020-73481-7

**Published:** 2020-10-05

**Authors:** Pyotr N. Petrov, Sergey E. Farisenkov, Alexey A. Polilov

**Affiliations:** Department of Entomology, Faculty of Biology, Lomonosov Moscow State University, Moscow, Russia

**Keywords:** Zoology, Entomology

## Abstract

Most microinsects have feather-like bristled wings, a state known as ptiloptery, but featherwing beetles (family Ptiliidae) are unique among winged microinsects in their ability to fold such wings. An asymmetrical wing folding pattern, found also in the phylogenetically related rove beetles (Staphylinidae), was ancestral for Ptiliidae. Using scanning electron, confocal laser scanning, and optical microscopy, high-speed video recording, and 3D reconstruction, we analyze in detail the symmetrical wing folding pattern and the mechanism of the folding and unfolding of the wings in *Acrotrichis sericans* (Coleoptera: Ptiliidae) and show how some of the smaller featherwing beetles have reverted to strict symmetry in their wing folding. The wings are folded in three phases by bending along four lines (with the help of wing folding patches on the abdominal tergites) and locked under the closed elytra; they unfold passively in two phases, apparently with the help of the elasticity provided by resilin unevenly distributed in the wing and of convexities forming in the cross-sections of the unfolding wing, making it stiffer. The minimum duration of folding is 3.5 s; unfolding is much more rapid (minimum duration lowest recorded in beetles, 0.038 s). The folding ratio of *A. sericans* is 3.31 (without setae), which is greater than in any beetle in which it has been measured. The symmetrical wing folding pattern found in *A. sericans* and in all of the smallest ptiliids, in which ptiloptery is especially pronounced, is the only known example of symmetry re-established during miniaturization. This direction of evolution is remarkable because miniaturization is known to result in various asymmetries, while in this case miniaturization was accompanied by reversal to symmetry, probably associated with the evolution of ptiloptery. Our results on the pattern and mechanisms of wing folding and unfolding can be used in robotics for developing miniature biomimetic robots: the mechanisms of wing folding and unfolding in Ptiliidae present a challenge to engineers who currently work at designing ever smaller flying robots and may eventually produce miniature robots with foldable wings.

## Introduction

Insects are the only group of flying invertebrates, and most insect species are winged and capable of flight. The earliest fossils of flying insects (Pterygota) are known from the Lower Carboniferous^1^ and predate the earliest flying vertebrates (Pterosaurida) by over 150 million years. Flight and wing folding were among the main reasons for the unprecedented evolutionary success of insects in terms of diversity^2,3^. Most flying insects have two pairs of wings, but in the majority of known pterygote species only one of these two pairs propels the insects in flight. Several lineages of the Pterygota have evolved various patterns of folding the wings of the pair used for flight^4^, based either on a zig-zag pattern consisting of subparallel folding lines or on an origami-like flexagon pattern consisting of oblique folding lines subdividing the wing blade into triangular facets^5^.

Beetles (Coleoptera) are among the few insect groups (others include Heteroptera and Dermaptera) that use only their hindwings for flight and keep them folded under the forewings (termed elytra in beetles) at rest^6^. The folding and unfolding of the wings have been studied in detail only in some species of beetles, but the folding patterns are at least generally similar in most of them^7–11^. At the start of folding, the unfolded wing is drawn posteriad so that it takes its resting position on the abdomen and the proximal portion of the wing is partly covered by the corresponding elytron; the wings are then folded under the elytra stepwise by a series of reciprocated brushing movements of the peculiarly microsculptured abdominal tergites^12–14^; at rest, the wings are locked under the elytra and thus prevented from unfolding^15^. Unfolding is more variable: even in the same beetle species, the wing can be unfolded either in the resting position or (more often) after it is drawn anteriad to take or approach its flight position^16^; the wing is then unfolded directly by its elasticity, largely provided by resilin^17–22^, or by spreading curved cross-sections, in the manner of a stiffening unfolded carpenter tape^14,23^, or by the hydraulic mechanism, increasing the pressure inside some of the major veins^24,25^, or (in many cases) apparently by two or all three of these mechanisms combined. The stiffness of the unfolded wing depends both on its composition and on its shape^26,27^.

Rove beetles (Staphylinidae) are among those relatively few groups of beetles in which the mechanisms of wing folding and unfolding have been studied in detail^28^. Rove beetles fold their wings asymmetrically and in very sophisticated flexagon-based patterns. These peculiar asymmetrical patterns result from simultaneous folding of overlapped wings, with the right and left crease patterns interchangeable, so that each wing can be folded in two different ways.

The Bilateria lineage, which comprises the vast majority of animals (Metazoa), including insects, is generally characterized by bilateral symmetry (reverted to radial symmetry in relatively few groups), but many of these bilaterally symmetrical organisms demonstrate directional left–right asymmetries, manifested in consistent heritable morphological differences between the left and right sides. It appears likely that these asymmetries are regulated by an ancestral mechanism, possibly involving cytoskeletal architecture^29^. Miniaturization, a major evolutionary trend among the Bilateria, tends to increase left–right asymmetries in various structures, such as the mouthparts, skeleton, nervous system, and reproductive system^30,31^.

The smallest free-living (non-parasitic) insects are featherwing beetles (Ptiliidae), a family phylogenetically close to Staphylinidae. Most of them are winged and have a peculiar wing shape found in most microinsects (less than 1 mm long), termed ptiloptery^32,33^: the wing blade is very narrow and “bristled” (fringed with relatively long peripheral setae). Ptiliids are unique among winged microinsects in their ability to fold such bristled wings.

In some of the less morphologically advanced ptiliids (the genera *Nossidium, Motschulskium,* and *Sindosium*), which comprise the subfamily Nossidiinae as recently defined^34,35^, the wings are folded asymmetrically^36,37^, in a flexagon pattern similar to that of Staphylinidae^28,37^. Surprisingly, the other ptiliids, which include the smallest species, fold their wings symmetrically, in fundamentally the same pattern, by bending them along four lines that are nearly perpendicular to the longitudinal axis of the wing and subparallel to each other (in the genus *Ptenidium* there is an additional fifth line of bending, not subparallel to the others). Thus, in most ptiliids the wing is folded in a symmetrical zig-zag pattern^37^. One of those many ptiliid species that fold their wings symmetrically is *Acrotrichis sericans*, the species that currently holds the record as the fastest (in terms of body lengths per second) of all ptiliids—and all flying animals^39^. The details of the mechanisms of the folding and unfolding of the wings in Ptiliidae have remained unknown.

Studying the unique patterns of the wing folding mechanisms of featherwing beetles has a potential application in robotics, the progress of which, like the evolution of the Bilateria, which tends to produce ever smaller organisms, tends to produce ever smaller robots, including flying ones^40–42^. At present all of these robots are much larger than the smallest free-living insects, but it is likely that the diminution of the robots will continue and may eventually come up to the challenge of designing miniature flying robots with foldable wings. Such robots could be used in many ways: the ability to fold and unfold their wings will help protect their wings when they are not used for flying. Work at mimicking the wing-folding of the larger beetles has already yielded some functioning models^43,44^.

It can be hypothesized that miniaturization results in peculiar changes not only in the structure of the wings but also in the patterns and mechanisms of wing folding and unfolding. The aim of this study was to test this hypothesis by analyzing the process of the surprisingly symmetrical folding and of the unfolding of the wings found in many ptiliids, using *Acrotrichis sericans* as a model.

## Results

### Folding pattern

**Figure 1:**
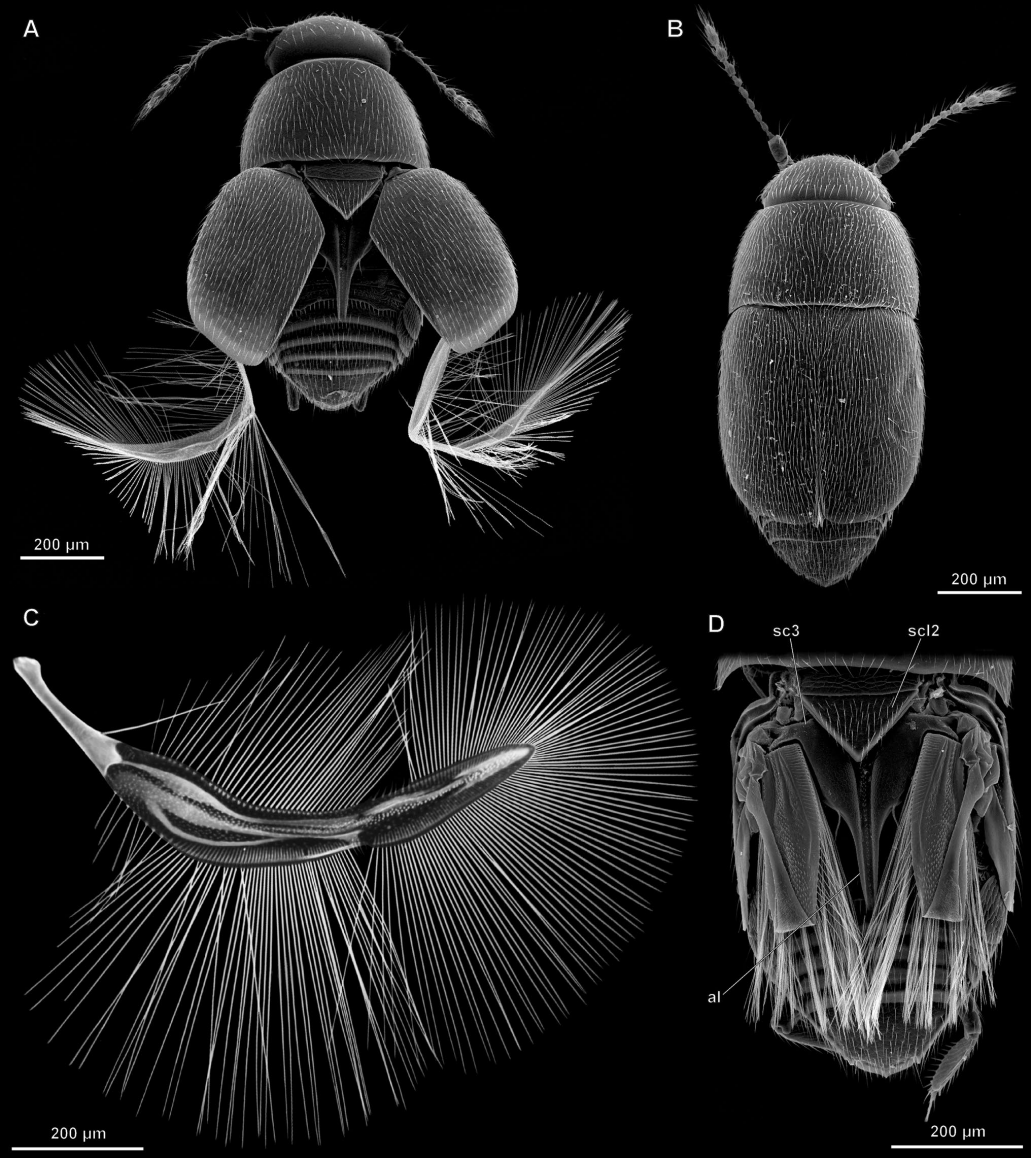
Wing folding of the featherwing beetle *Acrotrichis sericans*, SEM images (**A**,**B**,**D**) and optical microscope image (**C**): beetle with wings partly unfolded (**A**) and fully folded (**B**); unfolded wing (**C**) pterothorax and abdomen with wings fully folded, with elytra removed (**D**); *al* alacrista, *sc3* metascutum, *scl2* mesoscutellum.

*A. sericans* has wings about 1 mm long (for exact measurements, see^36^), approximately twice as long as the body (Fig. 1). The wings are folded at rest under the elytra, which are about half as long as the wings. Immediately before flight and during flight, the elytra are slightly raised and drawn apart, so that their sutural margins form an angle of about 60° (Fig. 1A). When the wings are at rest, the elytra are closed and locked together (Fig. 1B). The wings in *A. sericans*, as in all other winged representatives of the family Ptiliidae, are bristled, feather-like (Fig. 1C), narrow and divided into the shorter peduncle and the longer wing blade, which is surrounded by peripheral setae that are longer than the width of the wing blade. At rest, the wings are folded symmetrically under the elytra (Fig. 1D), by bending along four subparallel lines (and thus according to a zig-zag pattern), nearly perpendicular to the longitudinal axis of the wing, with the peripheral setae also folded, by twisting to some degree and bending at their bases, and directed posteriad.

### Resilin

**Figure 2:**
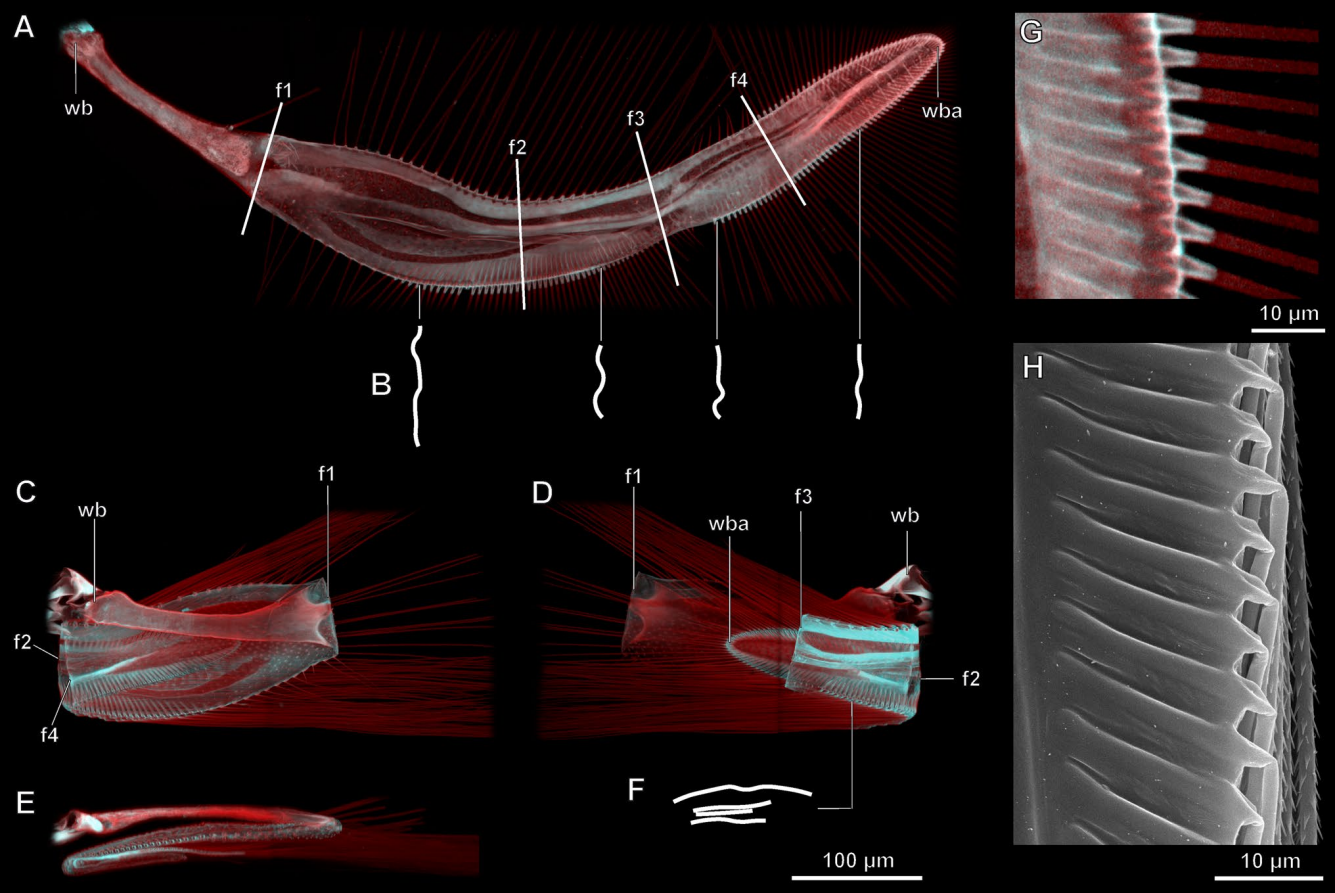
Structure of the wing of the featherwing beetle *Acrotrichis sericans*, confocal microscope images, autofluorescence from excitation at 405 and 559 nm (**A–G**) and SEM image (**H**): (**A**) fully unfolded wing in dorsal view, schematic profiles of cross-sections (obtained as optical sections of confocal stack) at marked points (**B**), fully folded wing in dorsal (**C**), ventral (**D**) and posterior view (**E**), and schematic profile of cross-section at marked point (**F**), margin of wing blade of fully unfolded wing, with setae also unfolded (**G**), margin of wing blade of fully folded wing with setae also folded (**H**); *wb* wing base, *wba* wing blade apex, *f1–f4* folds 1–4.

Resilin and sclerotization are distributed in the wing unevenly (areas colored blue and red, respectively, in Fig. 2). Resilin is concentrated in the area of the junction between the peduncle and wing blade, where the most proximal fold 1 is situated, and (especially broadly) in the area around and between the more distant folds 3 and 5 (with fold 4 in the middle between them); the rest of the wing is more sclerotized (Fig. 2A,C–E). Resilin is present in most areas of the wing blade, but there are no distinctly outlined areas with especially high concentrations of resilin, which can be due to the folding mechanism of the wing by reciprocating movements of the abdomen, see below, rather than by direct transverse folding). All setae are richer in resilin and less sclerotized at their bases (Fig. 2G), where they are folded at rest (Fig. 2H); the rest of each seta contains little resilin. Resilin is also present in the elongated sockets at the base of each seta (Fig. 2G), possibly contributing to the unfolding of the setae during the unfolding of the wings. The setae themselves (other than their bases) contain little resilin. The bases of the setae are hollow, and the setae are folded by slightly twisting at the bases, with the rest of the seta apparently rotating to a small degree relative to the base, and bending at a considerable angle relative to their erect position in unfolded wings.

### Wing profiles in cross-section

The wing profiles in cross-section are very different in the folded and in the unfolded states (Fig. 2B,F). In the folded state the wing blade is almost flat (Fig. 2F), while in the unfolded state it is more convex, with one or two waves that run along the wing blade subparallel to each other (Fig. 2B).

### Folding

**Figure 3:**
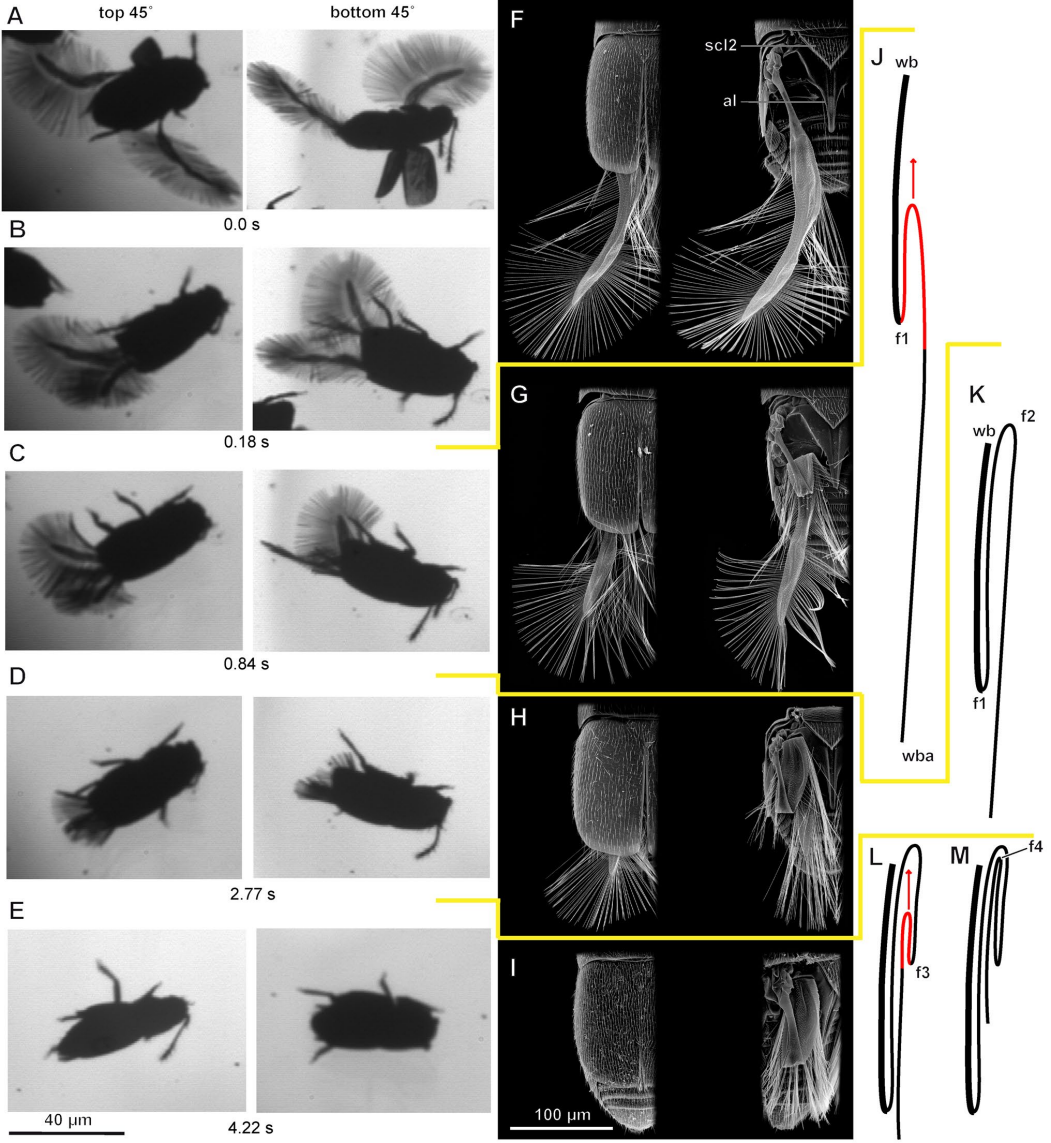
Wing folding process in the featherwing beetle *Acrotrichis sericans*: (**A–E**) Stages of wing folding in: frames of video recordings: wings drawn posteriad and then rotated (**A**); elytra closed (**B**); wings folded for the first time (**C**); wings folded for the second time (**D**); wings fully folded (**E**); top 45°, view from above at 45°; bottom 45°, view from below at 45°; (**F–I**) SEM images of pterothorax and abdomen in dorsal view with elytron intact (*left*) and removed (*right*); (**J–M**) wing profile in longitudinal section during stages 1–4 (**B–I**) of wing folding, area of wing with fold rolling along it and gradually changing its position (in contrast to conventional folding process, along lines nearly perpendicular to wing apex and under fully closed elytra) shown in red; *wb* wing base, *wba* wing blade apex, *f1–f4* folds 1–4, *al* alacrista, *scl2* mesoscutellum.

The process of wing folding in *A. sericans* includes three distinct phases (Fig. 3; Movie S1):

1. The wings are drawn posteriad and then rotated (Fig. 3A); the elytra are then closed (Fig. 3B,F).
2. Each wing is folded for the first time (folds 1 and 2: the former first, the latter at the end of this phase, Fig. 3C,G,J);
3. Each wing is folded for the second time (folds 3 and 4: also the former first, Fig. 3D,H,K, the latter at the end of this phase, Fig. 3E,I,L,M).

Fold 2 in phase 2 (Fig. 3J) and fold 4 in phase 2 are formed by tucking the more distal portion of the wing blade under the basal portion, with the location of the fold gradually shifting, so that the fold moves anteriad (Fig. 3L), completing the cycle with the zig-zag folding pattern maintained at rest (Fig. 3M).

The unfolded and folded wings of *A. sericans* measure as follows: length of unfolded wing with setae 1.02 ± 0.027 mm; length of unfolded wing without setae 0.83 ± 0.032 mm; length of folded wing with setae 0.402 ± 0.0011 mm; length of folded wing without setae 0.250 ± 0.0013 mm. The folding ratio of *A. sericans* is 2.53 with setae and 3.31 without setae.

The whole process of folding takes 3.57 to 6.10 s (4.51 ± 1.16 s). The first phase takes 0.176 ± 0.011 s; the second one takes 2.62 ± 0.63 s; the third one takes 1.71 ± 0.57 s. The first phase includes 7 to 10 reciprocating brushing movements of the abdomen (the distal portion of the abdomen bends ventrad with the abdomen elongating, then the distal portion is raised dorsad without the abdomen contracting, then the abdomen contracts, dragging the parts of the wings that touch the abdomen); the second phase includes 4 to 5 such movements.

**Figure 4:**
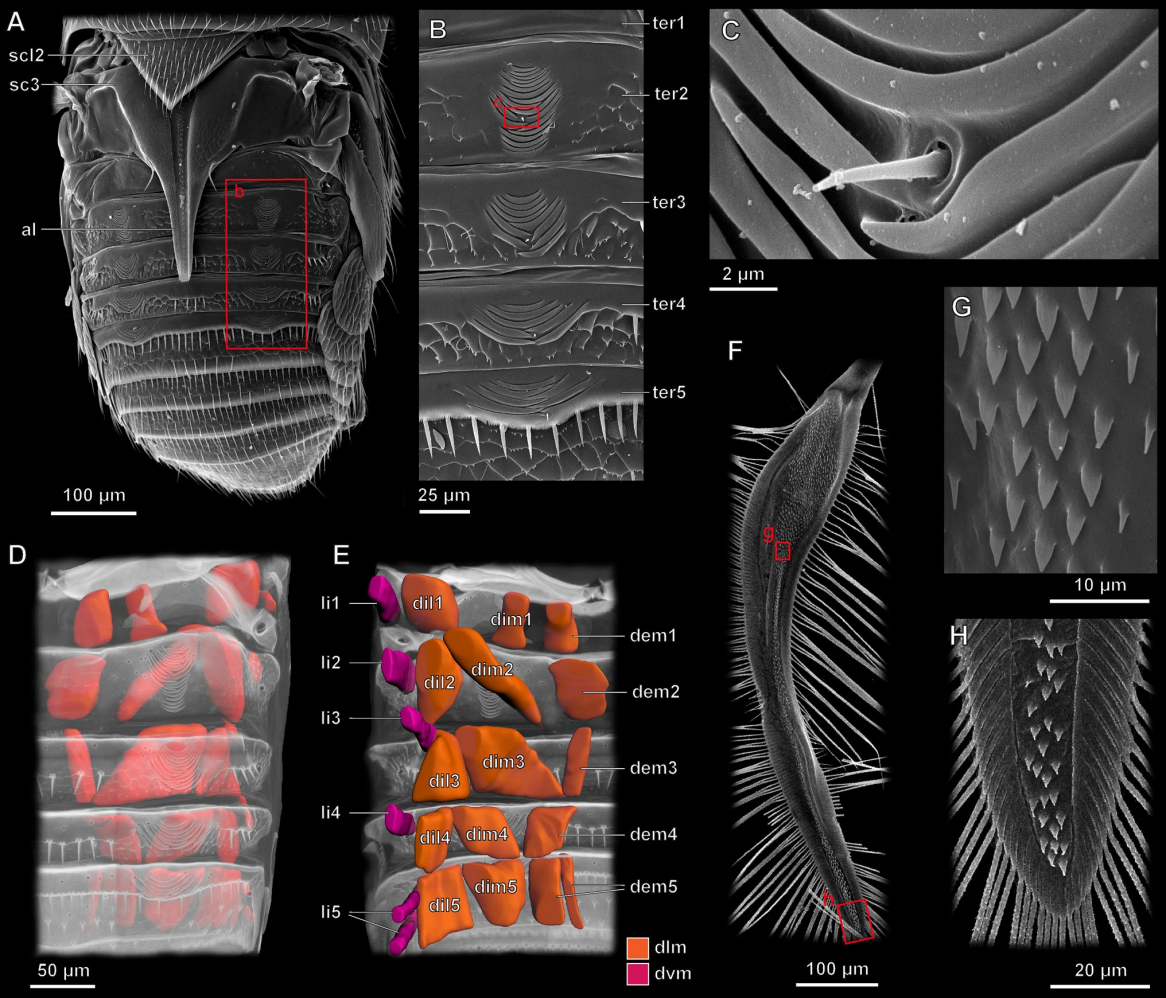
Wing folding mechanism of the featherwing beetle *Acrotrichis sericans*, SEM images (**A–C**,**F–H**) and 3D models (**D**,**E**): (**A**) abdomen and pterothorax; (**B**) close-up of sublateral fragment of abdominal tergites 1–5 showing wing-folding patches; (**C**) further close-up of sublateral fragment of wing-folding patch on abdominal tergite 2; 3D reconstruction of musculature in abdominal segments 1–5 in dorsal (**D**) and ventral (**E**) views; (**F**) wing blade in ventral view and close-up of medial (**G**) and apical (**H**) fragments (*dlm* dorsal longitudinal muscles, *dvm* dorsoventral muscles), *al* alacrista, *sc3* metascutum, *scl2* mesoscutellum, *ter1–5* abdominal tergites 1–5, *li1–5*, M. laterals interni of abdominal segments 1–5, *dil1–5* M. dorsales interni lateralis of abdominal segments 1–5, *dim1–5*, M. dorsales interni mediales of abdominal segments 1–5, *dem1–5* M. dorsales externi medialis of abdominal segments 1–5.

The folding of the wing is thus provided by movements of the abdomen. Peculiar wing folding patches on abdominal tergites 2–5 (Fig. 4A–C) are used to drag the wing by catching hold of triangular outgrowths on the medial area of the ventral surface of the wing blade (Fig. 4F–H). This sequence of movements of the abdomen is controlled by a system of dorsal longitudinal and dorsoventral muscles that create the waves of contractions running along the abdomen. Each tergite of the first five abdominal segments includes four pairs of muscles (Fig. 4D,E), three pairs of dorsal longitudinal muscles connecting the tergites (M. dorsales interni mediales, M. dorsales interni lateralis, and M. dorsales externi medialis (alternatively, this could be a subunit of M. dorsales interni mediales) and one pair of dorsoventral muscles connecting the tergite and the sternite (M. laterales interni).

Remarkably, the beetle can fold its wings while running.

### Unfolding

The unfolding of the wings is very rapid: it takes only from 0.038 to 0.063 s (0.053 ± 0.0094). First, folds 1 and 2 are straightened simultaneously (0.018 ± 0.00010 s); then folds 3 and 4 are straightened simultaneously (Movie S2).

At the beginning of the unfolding, the elytra are unlocked and start moving dorsad to their initial position at the start of flight, with the wings simultaneously unfolding and rather gradually and relatively slowly protruding more and more posteriad from under the elytra. The second and final phase of unfolding is more rapid: the wings fully unfold, protruding posterolaterad from under the elytra and then move posteriad, touching each other behind the abdomen.

## Discussion

### Folding pattern and folding ratio

The wing folding patterns of beetles are diverse and can be classified into many types and subtypes^7,9^. In most insects that can fold their wings, including the vast majority of beetles, longitudinal folds, more or less parallel with the stems of the main veins, are present^6,45^. As beetles evolved transverse wing folding, it made the folding apparatus and the wing as a whole more complex^11^. Transverse folding of the wings apparently played an important role in the evolution of beetles: most of the important hind wing synapomorphies that support the suborders of the Coleoptera are associated with transverse wing folding^45^. The folding patterns found in most Staphylinoidea (including the subfamily Nossidiinae of Ptiliidae, but excluding *Acrotrichis* and most other ptiliid genera) belong to the staphyliniform type^11^. Being relatively broad, the wing blades of typical staphylinoids are folded along several lines situated at different angles to each other (largely according to a flexagon pattern), mostly in the apical and central regions of the wing, but one of the folds (the jugal fold) is situated at the base.

According to the latest phylogenetic trees based on larval morphology^46^ and on adult morphology and molecular data^33^, the family Ptiliidae is a monophyletic group within the staphylinoid lineage, splitting into two clades, one of them comprising the subfamily Nossidinae and the other either divided into two subfamilies^46^ or comprising one subfamily Ptiliinae. We follow here the two subfamilies interpretation^33^. The folding pattern found in *A. sericans* (Fig. 2) is unique to Ptiliidae and found in nearly all (except the ptiliine genus *Ptenidium*, in which it is slightly modified by the emergence of an additional bend along an oblique folding line^36^) of the morphologically advanced genera of the family, i.e., all genera except the three ‘early splits’ that comprise the subfamily Nossidiinae (*Nossidium*, *Motschulskium*, and *Sindosium*), in which the folding pattern is very similar to that of Staphylinidae^34,37,38^. This is probably the ancestral folding pattern for Ptiliidae. Ptiliids of the subfamily Ptiliinae are unique among insects that can fold their wings in the absence of any longitudinal folds: all the folds are more or less transverse, relative to the longitudinal wing axis, and subparallel to each other^31^, in an ultimate manifestation of a zig-zag folding pattern (Fig. 3M). But the most unusual feature of this typical ptiliid pattern is its symmetry, which probably evolved in the common ancestor of the Ptiliinae during miniaturization, parallel to several other miniaturization-related features, which include pronounced ptiloptery (more pronounced in Ptiliinae than in Nossidiinae).

In addition to the folding patterns, foldable wings differ in the folding ratio, defined as the full length of the wing divided by the length of the folded wing, and therefore a measure of the degree to which the wing can shrink^23^. The folding ratio of those beetles in which it has been calculated varies between about 1.3 and about 2.5^23^. Ptiliids break this record: in *A. sericans* the folding ratio is 3.31 (without setae). If the length of the apical setae is included in the length of the wing, the ratio, although still very high, is 2.53, which is close to the highest values known in other beetles. It should be noted, however, that in all non-ptiliid beetles the apical setae on the wing margin are short in comparison with the length of the wing and the folding ratio probably usually implies the measured degree of the folding of the wing blade (the contribution of the apical setae being negligible). Therefore, it is likely that the degree to which the wing blades of *Acrotrichis* (and other ptiliines) shrink during folding is, indeed, the highest among beetles, and probably among any insects that fold their wings.

The emergence of the typical ptiliid wing folding pattern is especially surprising because it is probably the first known example of symmetry that evolved in an originally asymmetric structure during miniaturization. All other known examples include either retention of the original symmetry or emergence of asymmetry during miniaturization. It is therefore important to determine why this general rule is broken here. One possible explanation would be that the symmetrical folding pattern is an evolutionary consequence of increasing degrees of ptiloptery. In Staphylinidae and some other staphylinoids (including the subfamily Nossidiinae of Ptiliidae), the wing blade is relatively broad, and the asymmetry of folding probably results from the fact that the wings overlap under the relatively small elytra (extraordinarily small in most Staphylinidae as a result of their peculiar ecology^38^) at the early stages of folding, which renders their subsequent symmetrical folding difficult^28^. We hypothesize that as the wing blades grew narrower, they stopped overlapping during folding, and subsequent changes in the folding pattern that made it symmetrical could have been selected for because as evolution of ptiloptery made the peripheral setae of the wings relatively longer, their overlapping could complicate the folding process and compromise the mechanisms that control it.

### Process of folding

The process of wing folding varies between different groups of beetles, but it follows a common general sequence of movements^16,23,36^. The elytra are in many cases, including ladybird beetles^14^ and featherwing beetles, closed before wing folding. The muscle involved in the closing of each elytron is M. prophragma-mesophragmalis (IIdlm1, also known as M28^47^); this muscle is retained in Ptiliidae^12^. With the elytra fully or partly closed, the folding of the wings proceeds as follows. First, the base of the wing is rotated, possibly without active involvement of muscles, the rotation triggered by the collapse of axillary sclerite 3^16^, so that the wing acquires its resting position on the abdomen, remaining unfolded, and the corresponding elytron covers the proximal portion of the wing, with the distal portion protruding from under the elytra. Second, in most beetles the longitudinal folds are passively formed by relaxation of the wing^36^. This stage is missing in Ptiliidae, because their wing blades are too narrow to be folded longitudinally, and elastic properties of their wing blades are not involved in any longitudinal folding, in contrast to the vast majority of beetles. Then the wings are folded under the elytra step-wise by the combined action of elytra, the ventral surface of which is peculiarly microsculptured to perform this function, and a series of reciprocated anteriorly directed brushing movements of the abdominal tergites, which are densely covered with setae and other components of microsculpture^16,23^, using muscles of abdominal segments in combination with regulated changes in friction between various areas of the wing, abdominal tergites, and elytra: the microsculptures of all three components help to move the folding wing in only one direction, without allowing it to unfold after each phase of folding^13,36^.

The mechanisms involved in wing folding (other than the movements of the muscles that control the elytra and wings) can be classified into two categories, often working in combination: internal, relying on springs built in the wing itself, and external, depending on agencies other than the wing^36^. Folding in *Acrotrichis* is largely provided by movements of the abdomen, but such movements can vary, and advanced ptiliids, including *Arctotrichis*, differ from many other beetles in moving the abdomen, apart from its telescopic changes in length, only dorsad and ventrad, but not laterad, and in folding the wing exclusively with transverse folds, without any longitudinal ones. The recumbent denticles on the surface of the wing blade (Fig. 4F–H) in combination with the peculiar wing folding patches (Fig. 4A–C) help the abdomen to attach itself to the wing during the series of anteriad movements that allow the wing to be folded.

The movements of the abdomen are provided by a system of dorsal longitudinal and dorsoventral muscles (Fig. 4D,E). The musculature in Ptiliidae is partially reduced due to miniaturization^31,48^, and the muscles that participate in wing folding have apparently been preserved largely or mainly for this function. The evolutionary changes known in at least some representatives of the family include the loss of M. epimero-subalaris, M. furca-coxalis lateralis, M. furca-coxalis posterior^49^, and ventral muscles of the mesothorax^12^.

### Process of unfolding

The process of wing unfolding in beetles, even more variable than folding, also follows a common general sequence of movements, controlled by mechanisms different from those involved in folding^16^. The unfolding of the wings is triggered by the opening of the elytra. The opening movements of each elytron are facilitated by the following muscles: M. mesonoto-phragmalis, M. mesonoto-coxalis anterior, M. mesonoto-coxalis posterior, and M. mesepimero-subalaris (IIdlm2 IIdvm4, IIdvm5, and IItpm10, respectively; also known as M29, M40, M40, and M35, respectively^47^); all these muscles are retained in Ptiliidae^12^. The subsequent anteriad movement of each wing is facilitated by M. metanepisterno-axillaris and M. metepimero-axillaris tertius^16^: IIItpm7 and IIItpm 9, respectively; also known as M71b and M71a, respectively^47^); all these muscles are retained in Ptiliidae^12^.

Two mechanisms (other than the movements of the muscles), usually in combination, are involved in the unfolding of the wings^23^: the hydraulic mechanism and the release of stored elastic potential energy. Active (hydraulic) unfolding of the wings, as in some larger beetles^23–25^, is probably impossible in Ptiliidae, beetles of this family have no known pulsating organs^48–50^ and the remaining main veins in their wings do not have the typical tubular structure over most of their length^37^. Therefore, the unfolding of the wing is probably provided largely by the elasticity of the wing blade, and resilin probably contributes to the unfolding of the wing in combination with the convexities of the cross-section that spread longitudinally as the wing unfolds. The folded wings are locked under the elytra, and as soon as the elytra are unlocked, unfolding of the wings starts, and when the elytra are raised (to a greater angle than in larger beetles), the wings unfold rapidly, like a released spring. The subparallel waves stretched along the wing blade and visible in the cross-section of the unfolded wing (Fig. 2B) suggest a mechanism known also in other beetles: the unfolded wing could be rendered less flexible partly by curvature of the cross-section in the unfolded state, known as a carpenter tape^23^. This increased stiffness is probably useful for flight, and the carpenter tape mechanism is an elegant solution to make the wing suddenly stiffer during unfolding.

The waved cross-sections of the wing blade are presumably important both for flight and for wing folding and unfolding: making the wing stiffer, it allows the wing to be thinner at the same aerodynamic loads, thus decreasing the mass and the moment of inertia of the wing, a factor important for decreasing the inertial losses of the flight apparatus. In addition, folding a thicker wing blade to a small bending radius would either be impossible or require weaker sclerotization in the areas of the folds, which would have been detrimental to flight because of increasing the elastic deformations of the wing^27^.

The mechanism of unfolding is probably based exclusively on the elasticity of the materials of the wing. In the areas of the folds, the content of resilin is higher. Resilin is known to efficiently accumulate strain energy and unfold the cuticle to its initial shape. The role of resilin in wing unfolding has been shown also in other species of beetles^17–20,22^. Moreover, the cross-sections of the wing in the folded state become straighter, creating additional stress in the upper layers of the cuticle.

In contrast to the larger beetles and many other insects, in *Acrotrichis* resilin in the wing is diffused over relatively large areas (since venation is nearly absent), rather than localized in the junctures of the veins^17–20^. The relative concentration of resilin is higher mainly in the folding areas of the wing blade, but resilin is also present in the rest of the wing, probably facilitating the rolling shift of the folds during wing folding.

Only the basal portion of one vein in Ptiliidae contains a short cavity^37^. It is therefore unlikely that the hydraulic mechanism, largely responsible for unfolding the wings in some beetles, plays a major role in the mechanism of wing unfolding in Ptiliidae.

### Rates of folding and unfolding

The rates of folding and unfolding differ in *A. sericans* by two orders of magnitude (taking about 4.5 s vs. about 0.053 s, respectively).

The durations of wing folding recorded in other beetles are comparable: 1.38–3.83 s^13,28,51^. The highest and lowest previously recorded values of the duration of wing folding are 1.38 s in *Cafius vestitus*^28^ and 3.83 s in *Allomyrina dichotoma*^13^, respectively. In fact, most beetles can prolong wing folding duration almost indefinitely: under abnormal circumstances they sometimes do not complete wing folding. However, the maximum *normal* duration of wing folding recorded in *A. sericans* (6.10 s) is probably longer than that recorded in any other beeltee.

The duration of wing unfolding in *A. sericans* is, by contrast, extremely low (the minimum value in our observations is 0.038 s, lower than in any other beetle species in which this process has been recorded on video). The lowest previously recorded value of the duration of wing unfolding is 0.08 s in *Pachnoda marginata*, Scarabaeidae^16^. In beetles in general, the range of previously recorded durations of wing unfolding is 0.08–6.0 s^16,24,28,43,52,53^. The lowest duration of wing unfolding previously recorded in staphylinoid beetles is 0.1 s in *Cafius vestitus*, Staphylinidae^28^.

### Challenge for engineers

The results of our study may eventually contribute to designing biomorphic robots. Modern engineering is still far from producing miniature flying mechanisms comparable in size to the smallest insects, but the flying robots that are designed (e.g., the Robofly and RoboBee^41,42^) are becoming smaller and smaller, and engineers may soon be up to the challenge of creating an artificial model of a microbeetle. Eventually the wings of these microrobots could even become foldable—a feature that will open unique opportunities in using such flying apparatuses for various practical purposes, such as exploring previously inaccessible cavities, both natural and artificial.

## Materials and methods

### Material

Adults of the featherwing beetle *A. sericans* (Coleoptera: Ptiliidae) were collected at Zvenigorod Biological Station, Lomonosov Moscow State Univfersity (Moscow Oblast, Russia) in July and August 2019. Material for high-speed recording was delivered to the laboratory and kept under conditions close to natural for 1–2 days. Material for morphological studies was fixed in alcoholic Bouin solution or in ethanol and then stored in 70% ethanol.

### Scanning electron microscopy (SEM)

The fixed material was dehydrated in ethanol of increasing concentrations (80–95–100–100%), then in acetone (100%). The samples were critical point dried (Hitachi HCP-2) and sputter coated with gold (Giko IB-3) and then examined under a Jeol JSM-6380 scanning electron microscope at 20–30 kV with a working distance of 8 to 25 mm.

### Confocal laser scanning and optical microscopy

The fixed material was depigmented with a solution of hydrogen peroxide (Dimethyl sulfoxide + 100% EtOH + 30% H2O2 in proportions 1:3:1, respectively) for 1–5 days at a temperature of 37 °C, then dehydrated in ethanol of increasing concentrations (80–95–100–100) and cleared in BABB (Benzyl Alcohol + Benzyl Benzoate in proportions 1:2) for 24 h. After clearing, preparations were made in BABB between two coverslips with Teflon spacer rings. The samples were then studied and photographed under an Olympus FV10i-O confocal laser scanning microscope (CLSM) using 405 and 559 nm lasers. Unfolded wings were additionally photographed under an Olympus BX43 transmitted light microscope. Resilin was detected according to the principles developed by Michels and Gorb^21^ using material fixed in 70% ethanol.

### High-speed recording

High-speed video recording was performed using two synchronized Evercam 4000 cameras (Evercam, Russia) with a frequency of 250 fps (folding of the wings) to 4000 (unfolding), with a shutter speed of 3–30 μs in infrared light (LED 850 nm). The videos were recorded in chambers made of glass and aluminum at a temperature of 22–25 °C and a natural level of illumination in the visible spectrum in addition to the infrared light (invisible to the beetles) that we used for high-speed video recording. The cameras were placed at an angle of 90° to each other: one from above at 45° to the horizon, the other from below at the same angle. The recordings were analyzed using Fiji software package (ImageJ).

### 3D reconstruction

3D reconstruction of skeletal elements and muscles was performed on the basis of confocal stacks using the Bitplane Imaris program, in the “Surpass” module using the “Volume” and “Surfaces” functions. All structures were manually segmented using the latter function.

### Data analysis

Descriptive statistics were performed in R. Each measurement was replicated 10 times. Arithmetic mean ± SD is shown unless otherwise indicated.

## Supplementary information

Supplementary Legends.

Supplementary Video 1.

Supplementary Video 2.
