# Supplementary material for: Miniaturization re-establishes symmetry in the wing folding patterns of featherwing beetles
Source: Sci Rep. 2020 Oct 5;10:16458. doi: 10.1038/s41598-020-73481-7 (PMC7536412; doi:10.1038/s41598-020-73481-7)
Supplement: Supplementary file 1 — Supplementary Legends. [file 41598_2020_73481_MOESM1_ESM.docx]

**Miniaturization re-establishes symmetry in the wing folding patterns of featherwing beetles**

**Pyotr N. Petrov, Sergey E. Farisenkov, Alexey A. Polilov^*^**

Department of Entomology, Faculty of Biology, Lomonosov Moscow State University, Moscow, Russia

**Supplementary information**

**Movie S1** (separate file). Wing folding of the featherwing beetle *Acrotrichis sericans* (high-speed video recording).

**Movie S2** (separate file). Wing unfolding of the featherwing beetle *Acrotrichis sericans* (high-speed video recording).
